# Increased perceived stress is negatively associated with activities of daily living and subjective quality of life in younger, middle, and older autistic adults

**DOI:** 10.1002/aur.2779

**Published:** 2022-07-05

**Authors:** Goldie A. McQuaid, Colin H. Weiss, Alex Job Said, Kevin A. Pelphrey, Nancy Raitano Lee, Gregory L. Wallace

**Affiliations:** ^1^ Department of Psychology George Mason University Fairfax Virginia USA; ^2^ Department of Speech, Language, and Hearing Sciences The George Washington University Washington District of Columbia USA; ^3^ Department of Neurology University of Virginia School of Medicine Charlottesville Virginia USA; ^4^ Department of Psychological and Brain Sciences Drexel University Philadelphia Pennsylvania USA

**Keywords:** activities of daily living, adulthood, autism, perceived stress, subjective quality of life

## Abstract

Few studies have examined self‐reported perceived stress in autistic adults. Existing studies have included relatively small, predominantly male samples and have not included older autistic adults. Using a large autistic sample (*N* = 713), enriched for individuals designated female at birth (59.3%), and spanning younger, middle, and older adulthood, we examined perceived stress and its associations with independence in activities of daily living and subjective quality of life (QoL). Perceived stress for autistic adults designated male or female at birth was compared to their same birth‐sex counterparts in a general population sample. In addition, within the autistic sample, effects of sex designated at birth, age, and their interaction were examined. Regression modeling examined associations between perceived stress and independence in activities of daily living and domains of subjective QoL in autistic adults, after controlling for age, sex designated at birth, and household income. Autistic adults reported significantly greater perceived stress than a general population comparison sample. Relative to autistic adults designated male at birth, those designated female at birth demonstrated significantly elevated perceived stress. Perceived stress contributed significantly to all regression models, with greater perceived stress associated with less independence in activities of daily living, and poorer subjective QoL across all domains—Physical, Psychological, Social, Environment, and Autism‐related QoL. Findings are contextualized within the literature documenting that autistic individuals experience elevated underemployment and unemployment, heightened rates of adverse life events, and increased exposure to minority stress.

**Lay Summary:**

This study looked at self‐reported perceived stress in a large sample of autistic adults. Autistic adults reported more perceived stress than non‐autistic adults. Autistic individuals designated female at birth reported higher stress than autistic individuals designated male at birth. In autistic adults, greater perceived stress is related to less independence in activities of daily living and poorer subjective quality of life.

## INTRODUCTION

Stress is implicated in an array of poor health outcomes (Thoits, 2010). Detrimental impacts of stress on health include immune system dysfunction (Glaser & Kiecolt‐Glaser, 2005), cardiovascular disease (Kivimäki & Steptoe, 2018), depression (Slavich & Irwin, 2014), and anxiety (Juruena et al., 2020). Stress can be operationalized in different ways. Stress may be measured via biological assays, such as levels of skin conductance or the stress hormone cortisol. Alternatively, stress may be defined by life experiences (e.g., having experienced a natural disaster or a major negative life event such as the death of a loved one or loss of a job and income) or by the life‐roles one assumes (e.g., pregnancy‐related stress, caregiver stress). Further, stress may be quantified based, not on a particular event or role per se, but instead based on an individual's report of how stressful they evaluate the relevant event or role to be.

There is a growing literature on the impacts of stress in autism spectrum disorder (ASD); however, studies have employed different operationalizations of stress. A number of studies have examined self‐reported experiences of stress and have demonstrated elevated stress in autistic individuals. For instance, studies have shown autistic adults report elevated stress assessed via the stress subscale of the Depression, Anxiety Stress Scales (DASS; Henry & Crawford, 2005; S. H. Lovibond & Lovibond, 1995) (George & Stokes, 2018; Park et al., 2019), a metric that more nearly approximates generalized anxiety, as opposed to stress (P. F. Lovibond, n.d.). Other studies have used the Stress Survey Schedule (SSS; Groden et al., 2001), asking autistic individuals to rate the level of stress they would experience when encountering certain events (e.g., being near bright lights, waiting in line, having a conversation). These studies have shown autistic females report higher levels of stress in response to events involving sensory stimuli and personal contact, relative to autistic males, and older autistic adults report greater stress than their younger counterparts for all domains except exposure to sensory stimuli and personal contact (Gillott & Standen, 2007; McGillivray & Evert, 2014). Research has also examined lifetime exposure to major stressors and perceived severity of those stressors using the Stress and Adversity Inventory for adults (STRAIN; Slavich & Shields, 2018), showing autistic adults reported greater stress responsivity overall, and in particular greater severity of stress in response to stressors characterized by change, humiliation, and physical danger (Moseley et al., 2021).

Unlike the self‐reported measures of stress used above, stress may also be defined more broadly, without reference to or consideration of any particular life event or role and without inquiring about specific experiences. This more global measure of stress queries an individual's appraisal of how stressful their everyday life is and how equipped they assess themselves to be to handle that everyday stress. This last conceptualization of stress is referred to as perceived stress. Perceived stress may be a particularly important metric of stress to employ in understanding the experiences and impacts of stress in ASD, given that relative to neurotypical individuals, autistic individuals may experience everyday life differently and may therefore identify different stimuli and events as stressors. For example, the core features of ASD include challenges in navigating social situations, sensory processing differences, and discomfort with transitions and novel situations. Thus, as a metric of stress, perceived stress allows for research that privileges the global autistic experience of stress, rather than presuming that certain stimuli or events may be sources of stress for autistic individuals.

Literature examining perceived stress to date has shown that compared to neurotypical adults, autistic adults report higher levels of perceived stress (Bishop‐Fitzpatrick, Minshew, et al., 2017; Hirvikoski & Blomqvist, 2014; McLean et al., 2021), and higher levels of perceived stress in autistic adults are associated with reduced social functioning (Bishop‐Fitzpatrick, Smith DaWalt, et al., 2017). Furthermore, greater perceived stress is linked to poorer subjective QoL (Bishop‐Fitzpatrick, Smith DaWalt, et al., 2017) in autistic adults, unlike neurotypical adults, when controlling for age, sex, and IQ (Bishop‐Fitzpatrick et al., 2018). Additionally, higher levels of perceived stress in autistic adults increased the association between poor sleep quality and diminished subjective quality of life (McLean et al., 2021). It is important to note that two of the aforementioned studies (Bishop‐Fitzpatrick et al., 2018; Bishop‐Fitzpatrick, Minshew, et al., 2017) recruited participants from an ongoing trial of a psychosocial intervention. As such the autistic participants in these studies may not be representative of the broader population of autistic adults. Finally, one study examining a small sample of autistic adults (*N* = 60; autistic female: *n* = 14) found sex differences in perceived stress in autistic adults, with autistic females reporting elevated levels of perceived stress relative to autistic males (Hong et al., 2016), a finding that parallels sex differences in perceived stress reported for the general population (Cohen & Janicki‐Deverts, 2012). For details on prior empirical studies that have examined perceived stress in autistic adult samples, see Table 1.

**TABLE 1:** Summary of empirical literature implementing self‐report on the Perceived Stress Scale (PSS) in autistic adult samples

Study	Participant groups, *ns*	Age, years mean (SD), range	Sex designated at birth	Aims/research questions	PSS version	Variables examined for relationships with PSS	Group differences	Relationships of other variables with perceived stress
Hirvikoski and Blomqvist (2014)	ASD = 25 NT = 28	ASD 34.08 (7.52) NT 32.64 (6.99)	ASD Female: 10 Male: 15 NT Female: 16 Male: 12	Examine perceived stress in ASD & associations between autistic features and facets of perceived stress	14‐item PSS	Autism Spectrum Quotient (AQ)	Total PSS: ASD > NT PSS Distress subscale: ASD > NT PSS Coping subscale: ASD > NT	‐ Higher AQ score associated with greater total PSS score in ASD and NT ‐ Higher AQ score correlated with PSS Distress and PSS Coping subscale scores in ASD and NT
Hong et al. (2016)	ASD = 60	ASD 32 (6.8) 25–55	ASD Female: 14 Male: 46	Probe factors associated with subjective QoL	10‐item PSS	WHOQOL‐BREF	N/A	Perceived stress: Female > Male ‐ Perceived stress predicted all domains of subjective QoL, with higher perceived stress associated with lower subjective QoL
Bishop‐Fitzpatrick, Minshew, et al. (2017)^a^	ASD = 40 (ASD participants recruited from an active intervention study) NT = 25	ASD 24.20 (6.95) 18–44 NT 24.84 (3.69) 18–32	ASD Female: 4 Male: 36 NT Female: 4 Male: 21	Examine whether poor response to stress negatively impacts social functioning in ASD	10‐item PSS	W‐ADL Social Adjustment Scale‐II (SAS‐II)	Perceived stress: ASD > NT	‐ Greater perceived stress associated with greater social disability in ASD, as measured by the SAS‐II ‐ Perceived stress was not significantly associated with independence in activities of daily living, as assessed via the W‐ADL
Bishop‐Fitzpatrick, Smith DaWalt, et al. (2017)	ASD = 67	ASD 31.5 (6.7) 24–55	ASD Female: 21 Male: 46	‐ Investigate association of perceived stress with subjective QoL ‐ Probe whether social and recreational activities moderate association between perceived stress and subjective QoL	10‐item PSS	WHOQOL‐BREF Social and recreational activities	N/A	‐ Greater perceived stress associated with lower subjective QoL ‐ Recreational activities, but not social activities, moderated effects of perceived stress on subjective QoL
Bishop‐Fitzpatrick et al. (2018)^a^	ASD = 40 (ASD participants recruited from an active intervention study) NT = 25	ASD 24.20 (6.95) 18–44 NT 24.84 (3.69) 18–32	ASD Female: 4 Male: 36 NT Female: 4 Male: 21	‐ Investigate impacts of perceived stress on subjective QoL ‐ Examine whether social support serves as a buffer in this association	10‐item PSS	WHOQOL‐BREF Interpersonal Support Evaluation List (ISEL)	Perceived stress: ASD > NT	‐ Higher perceived stress associated with lower subjective QoL in ASD but not NT ‐ Social support did not moderate effect of perceived stress on subjective QoL for ASD or NT
Pahnke et al. (2019)	ASD = 10	ASD 49 (12), 25–65	ASD Female: 5 Male: 5	Pilot study to determine feasibility of acceptance and commitment therapy (ACT) with ASD adults and to assess its effects on perceived stress	14‐item PSS	Effect of intervention examined: comparison of PSS at baseline, post‐treatment and 3 months follow‐up	N/A	‐ Significant reduction in perceived stress from baseline to post‐treatment ‐ No significant difference in perceived stress when comparing baseline to 3 months follow‐up
Wijker et al. (2020)	ASD = 53 Intervention group = 27 Control condition = 26	ASD 18–60	ASD Female: 23 Male: 29	Randomized control trial exploring effects of animal assisted therapy (AAT) compared to waitlist condition on perceived stress and other variables in ASD adults	10‐item PSS	AAT intervention or control condition	N/A	Those receiving AAT (but not those in control group) showed significant reduction in perceived stress from baseline to post‐intervention
McLean et al. (2021)	ASD = 40 NT = 24	ASD 24.2 (6.95) NT 25 (3.68)	ASD Female: 4 Male: 36 NT Female: 4 Male: 20	‐ Examine effects of perceived stress on subjective QoL in ASD ‐ Explore whether being autistic moderates associations between perceived stress and sleep quality with subjective QoL	10‐item PSS	WHOQOL‐BREF Pittsburgh Sleep Quality Index (PSQI)	Perceived stress: ASD > NT	‐ Higher perceived stress was associated with lower subjective QoL ‐ No interaction between perceived stress and group (ASD, NT) on subjective QoL ‐ Compared to NT, ASD with high perceived stress and poor sleep quality reported worse subjective QoL

Abbreviations: NT, neurotypical; W‐ADL, Waisman‐Activities of Daily Living; WHOQOL‐BREF, Brief version of the World Health Organization Quality of Life Scale.

^a^ The studies of Bishop‐Fitzpatrick, Minshew, et al. (2017) and Bishop‐Fitzpatrick et al. (2018) report on the same participants.

A small body of research suggests perceived stress may serve as a target for therapeutic intervention in autistic adults. A randomized controlled trial showed that, compared to autistic adults assigned to a waitlist condition, those who received dog‐assisted therapy reported decreased perceived stress at follow‐up timepoints relative to baseline (Wijker et al., 2020), and autistic adults assessed before and after a 12‐week intervention utilizing acceptance and commitment therapy reported a significant reduction in perceived stress (Pahnke et al., 2019).

To advance our understanding of perceived stress in autistic adults, the current study examined potential relationships between perceived stress and activities of daily living and subjective quality of life (QoL) in a large sample of autistic adults spanning young, middle, and older adulthood. We sought to replicate findings in the extant literature on perceived stress that have documented higher perceived stress in autistic adults relative to non‐autistic adults, and that have shown elevated perceived stress in autistic adults is negatively associated with overall subjective QoL. We also sought to augment the existing literature, examining potential links between perceived stress and activities of daily living and subjective QoL across multiple domains—Physical Health, Psychological Health, Environment, Social Relationships, and Autism‐related QoL—while controlling for key, potentially confounding, variables: sex designated at birth, age, and a metric of socioeconomic status.

We hypothesized that autistic adults would show elevated perceived stress relative to a comparison general population sample, and that autistic adults designated female at birth would report greater levels of perceived stress relative to autistic adults designated male at birth. Because sex designated at birth (Cohen & Janicki‐Deverts, 2012; Cohen & Williamson, 1988; Xu et al., 2015), age (Cohen & Janicki‐Deverts, 2012; Cohen & Williamson, 1988; Nordin & Nordin, 2013; Osmanovic‐Thunström et al., 2015), and socioeconomic status (Cohen & Janicki‐Deverts, 2012; Redmond et al., 2013) have been associated with perceived stress, these variables were accounted for in the analyses examining relations between perceived stress and the key outcomes of interest: activities of daily living and subjective QoL. Therefore we predicted that after accounting for effects of sex designated at birth, age, and socioeconomic status, heightened levels of perceived stress would predict lower independence in activities of daily living, and poorer subjective QoL across all measured domains in autistic adults.

## METHODS

### Participants

Participants were recruited via Simons Powering Autism Research and Knowledge (SPARK; The SPARK Consortium, 2018) Research Match. All participants took part in a broader online study of adult development and outcomes in ASD and were provided $25 for completing study procedures. Seven hundred and thirteen adults (59.3% female) ranging in age from 18.17 to 83.33 years (*M* = 38.47, SD = 13.60) were included in the current analyses. All data reported on here were collected between December 2019 and mid‐January 2020, prior to the first laboratory‐confirmed case of COVID in the United States.

The sample was composed of “independent” autistic adults, as designated by SPARK. “Independent adults” are individuals ≥18 years of age who do not have a court‐appointed legal guardian and who can therefore consent for themselves. Given these SPARK criteria for “independent adult” status, participants were unlikely to have a co‐occurring intellectual disability. Additionally, as part of a medical history questionnaire collected in the current study, no participant reported intellectual disability as a past or current diagnosis.

To be included in analyses here, a self‐disclosed community‐based diagnosis of an autism spectrum disorder provided by a medical/clinical professional was required. SPARK does not independently confirm diagnoses; however, SPARK partners with and recruits from expert autism clinical sites, in part, to increase the likelihood that participants have a professional autism spectrum diagnosis (The SPARK Consortium, 2018). Additionally, a study examining a sample of 254 SPARK participants, including “independent” adults, confirmed an autism spectrum diagnosis in 98.8% of the sample using electronic medical records (Fombonne et al., 2021). The study concluded that the validity of disclosed autism spectrum diagnoses, including self‐disclosed diagnoses, were independently confirmed with “high confidence” (Fombonne et al., 2021). Consistent with the self‐disclosed clinical diagnoses of participants in the current study, 94.5% of the sample (*n* = 674) met screening criteria (total scores of >65) on the Autism Spectrum Quotient‐Short Form (AQ‐28; Hoekstra et al., 2011).

Demographic characteristics of the sample are presented in Table 2. The study was approved by The George Washington University Institutional Review board and followed procedures in accordance with the Declaration of Helsinki.

**TABLE 2:** Participant characteristics and mean self‐report ratings

Construct	*N* = 713
Age, years	
Mean (SD)	38.47 (13.60)
Median (range)	36.08 (18.17–83.33)
Sex designated at birth, *n* (%)	
Female	423 (59.3%)
Male	290 (40.7%)
Gender identity^a^	
Gender diverse	74 (10.4%)
Cisgender	638 (89.6%)
Race and ethnicity, *n* (%)	
Race^a^	
Asian	9 (1.3%)
Black/African‐American	15 (2.1%)
More than one race	70 (9.8%)
Native American/Alaska Native	8 (1.1%)
White	594 (83.7%)
Other	14 (2.0%)
Ethnicity^a^	
Latinx	63 (8.9%)
Not Latinx	634 (89.5%)
Unknown	11 (1.6%)
Household Income, *n* (%)	
<$20,000	257 (36.0%)
$20,001–$35,000	132 (18.5%)
$36,000–$50,000	96 (13.5%)
$51,000–$65,000	39 (5.5%)
$66,000–$80,000	56 (7.9%)
$81,000–$100,000	38 (5.3%)
$101,000–$130,000	37 (5.2%)
$131,000–$160,000	26 (3.6%)
$161,000+	32 (4.5%)
Current employment and/or educational enrollment,^a^ *n* (%)	
Currently employed, not enrolled in educational program	307 (43.4%)
Currently enrolled in educational program, not employed	57 (8.1%)
Currently employed and enrolled in educational program	62 (8.8%)
Not employed or enrolled in educational program	281 (39.7%)
Autism spectrum quotient‐28, total score	
Mean (SD)	84.32 (11.58)
Median (Range)	85.0 (47–112)
Met autism spectrum quotient‐28 screening criteria, *n* (%)	
Yes	674 (94.5%)
No	39 (5.5%)
Perceived Stress Scale, total score	
Mean (SD)	22.81 (7.42)
Median (Range)	23.0 (1–40)
Waisman Activities of Daily Living, item score	
Mean (SD)	1.78 (0.28)
Median (Range)	1.88 (0–2)
WHOQOL‐BREF‐Physical Health, item score	
Mean (SD)	3.29 (0.87)
Median (range)	3.33 (1–5)
WHOQOL‐BREF‐Psychological Health, item score	
Mean (SD)	2.96 (0.84)
Median (range)	3.0 (1–5)
WHOQOL‐BREF‐Social Relationships, item score	
Mean (SD)	3.01 (1.01)
Median (range)	3.0 (1–5)
WHOQOL‐BREF‐Environment, item score	
Mean (SD)	3.46 (0.81)
Median (range)	3.46 (1–5)
Autism Specific Quality of Life, item score	
Mean (SD)	3.11 (0.84)
Median (range)	3.12 (1–5)

Abbreviation: WHOQOL‐BREF, World Health Organization Quality of Life Questionnaire.

^a^ Gender identity, *N* = 712; Race, *N* = 710; Ethnicity, *N* = 708; Complete data for both currently enrolled in educational program and currently employed, *N* = 707.

## MEASURES

### Perceived stress

The Perceived Stress Scale (PSS; Cohen et al., 1983; Cohen & Williamson, 1988) is one of the most commonly used instruments for the measurement of perceived stress, and has been implemented in neurotypical (Ezzati et al., 2014; Tan et al., 2020) and autistic samples (Bishop‐Fitzpatrick et al., 2018; Bishop‐Fitzpatrick, Minshew, et al., 2017; Wijker et al., 2020).

In its initial deployment, the PSS was comprised of 14 items (Cohen et al., 1983). The 14‐item scale showed adequate reliability across three samples (Cronbach's alpha = 0.84–0.86). The measure was also correlated with life‐event impact scores across those samples (*r* = 0.24–0.49), which to some degree assesses the subjective assessment of stress, and therefore supports the construct validity of the PSS (Cohen et al., 1983). Moreover, the PSS was a better predictor of health‐related outcomes than were life‐event scores, which probed the number of negative life events (derived from the Unpleasant Events Schedule [Lewinsohn & Talkington, 1979]), and the respondents' rating regarding the impact of the relevant aversive events.

A modified version of the PSS, comprised of 10 items (Cohen & Williamson, 1988), has shown reliability and validity comparable to the 14‐item PSS (Roberti et al., 2006). The current study employed the 10‐item PSS, which forms a unidimensional scale of global perceived stress including questions that probe how stressed the respondent has felt over the past month and how confident they have felt in terms of handling that stress (e.g., “In the last month, how often have you felt difficulties were piling up so high that you could not overcome them?”). Broadly, across different samples, the 10‐item PSS demonstrates good internal consistency reliability (Cronbach's alpha = 0.78–0.91) (Cohen & Janicki‐Deverts, 2012; Cohen & Williamson, 1988). Further, and of particular relevance to the current study, within a sample of autistic adults without co‐occurring ID, the PSS has shown good internal consistency reliability (Cronbach's alpha = 0.87) (Bishop‐Fitzpatrick, Minshew, et al., 2017).

Participants responded to each item on a five‐point Likert scale (ranging from 0 = Never to 4 = Very Often). Total scores range from 0 to 40, with higher scores indicating greater levels of perceived stress. In the current study, Cronbach's alpha for the PSS was 0.89. In all analyses, the PSS was used as the independent variable.

### Activities of daily living

Participants' independence in the performance of activities of daily living was assessed using the Waisman Activities of Daily Living (W‐ADL; Maenner et al., 2013). The W‐ADL is comprised of 17‐items answered on a three‐point Likert scale (0 = Does not do at all; 1 = Does with help; 2 = Independent, or does on own). W‐ADL items were summed to generate a total score ranging from 0 to 34, with higher scores indicative of greater independence in activities of daily living. The W‐ADL has high internal reliability (Cronbach's alpha = 0.81; Bishop‐Fitzpatrick, Minshew, et al., 2017) and has demonstrated robust reliability over time, with weighted kappas ranging from 0.92 to 0.93 (Maenner et al., 2013). The W‐ADL mean item score served as a dependent variable in analyses reported here.

### Subjective quality of life

Subjective quality of life (QoL) was assessed via the Abbreviated World Health Organization Quality of Life (WHOQOL‐BREF; The WHOQOL Group, 1998), and the Autism Specific Quality of Life (ASQoL; McConachie et al., 2018) questionnaires. The WHOQOL‐BREF is a 26‐item assessment focusing on four domains of subjective QoL: Physical Health (seven items), Psychological Health (six items), Social Relationships (three items), and Environment (eight items). Two additional items query general health and global QoL. Participants responded to each item on a five‐point Likert scale. A score was generated for each of the four domains by summing respective item responses. Higher scores reflect better subjective QoL. Analyses reported here used the mean item score for each of the four domains (range 1–5) as dependent variables.

The ASQoL was developed through engagement with autistic adults to formulate “autism‐specific” subjective QoL items to be administered in conjunction with the WHOQOL‐BREF (McConachie et al., 2018). The ASQoL consists of nine items answered on a five‐point Likert scale (1 = Not at All/Never, 5 = Totally/Always). An ASQoL score is calculated by averaging responses to the initial eight survey items. A final ninth item, which asks about “autistic identity,” is not included in the calculation of the ASQoL score. Mean scores range from 1 to 5, with higher scores reflecting better subjective QoL. In analyses reported here, the ASQoL mean score was used as a dependent variable.

### Data analysis

To contextualize perceived stress in the current sample of autistic adults, we compared mean PSS scores in our sample to those scores collected from a large sample of adults from the general population. In 2009, the 10‐item PSS was administered to 4000 adults in the United States (Cohen & Janicki‐Deverts, 2012). Using two one‐sample *z*‐tests, we compared PSS mean scores in autistic females and males in the current sample with those of females and males, respectively, in the aforementioned general population sample.

To evaluate potential sources of perceived stress heterogeneity within the autistic sample, a 2 × 2 analysis of variance (ANOVA) examined the effects of sex designated at birth (female vs. male), age group (younger [18–39 years; *n* = 421] vs. older [40–83 years; *n* = 292] adults) and a sex × age interaction.

To probe relationships between perceived stress and the outcomes of interest, multiple linear regression analyses were employed. These analyses investigated the contributions of perceived stress to subjective QoL (WHOQOL‐BREF Physical Health, Psychological Health, Social Relationships, and Environment domain scores; ASQoL total score) and daily living skills (W‐ADL total score) after accounting for potentially influential covariates, including age, annual household income, and sex designated at birth. Additionally, the regression between perceived stress and psychological QoL was rerun omitting one of the items from the Psychological Health QoL domain due to its content overlap with an item on the PSS. Details of this analysis and its results are reported in  S1. Corrections for multiple comparisons in the regression models were made using the Bonferroni method, and regression results surviving the adjusted *p*‐value (0.05/6 = 0.0083) were considered statistically significant.

## RESULTS

**FIGURE 1:**
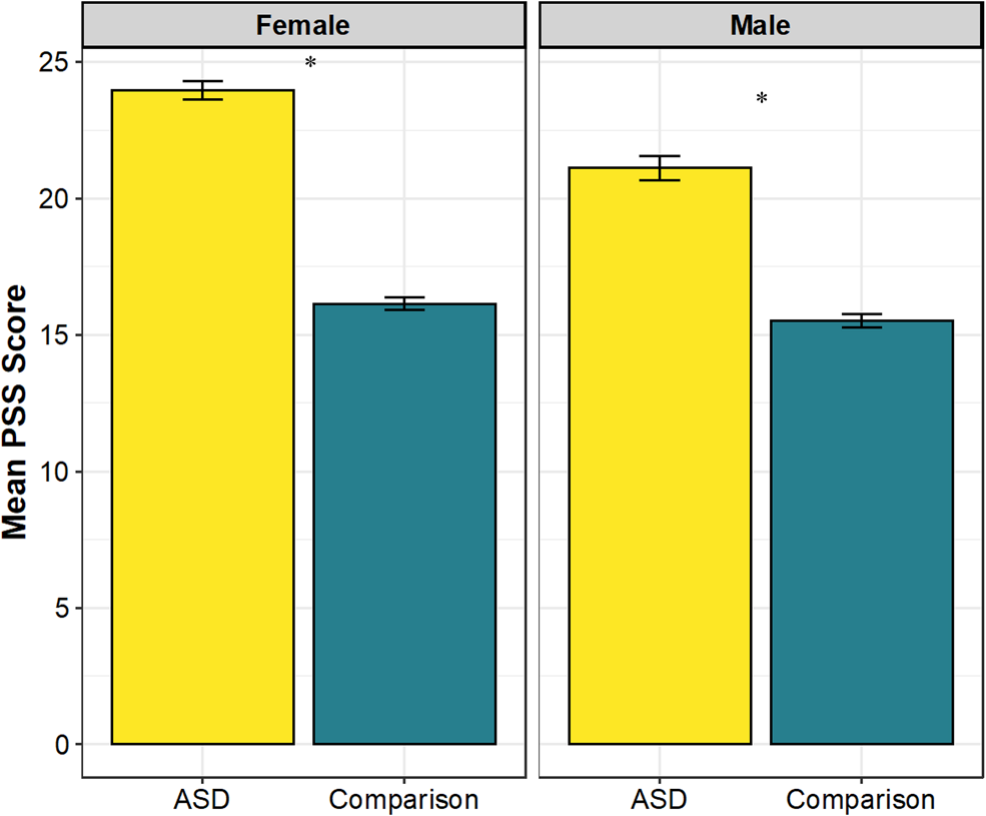
Mean 10‐item Perceived Stress Scale (PSS) scores in current ASD sample and comparison general population data (Cohen & Janicki‐Deverts, 2012), by sex designated at birth. Error bars represent standard error of the mean. *p < 0.0001

One‐sample *z*‐tests revealed that compared to the general population adult females (*M* = 16.14, SD = 7.56) and males (*M* = 15.5, SD = 7.44), autistic females (*M* = 23.96, SD = 7.07) and males (*M* = 21.13, SD = 7.61) reported significantly elevated levels of perceived stress (females: *z* = 6.18, *p* < 0.0001; males: *z* = 4.19, *p* < 0.0001). See Figure 1.

**FIGURE 2:**
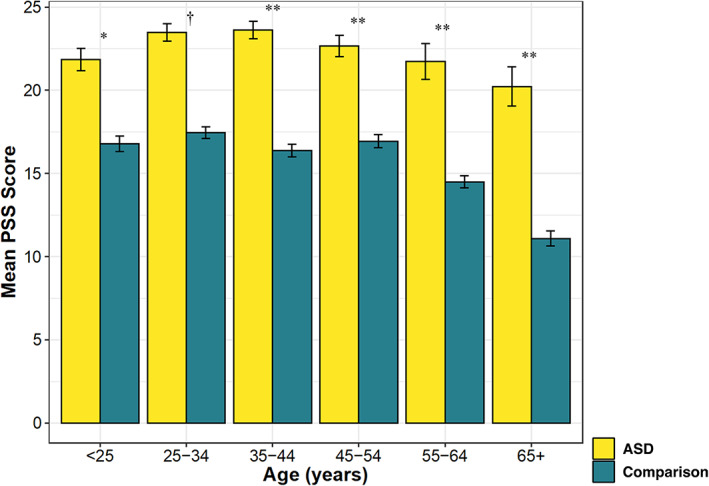
Post‐hoc group differences analyses for mean 10‐item Perceived Stress Scale (PSS) scores in current ASD sample and comparison general population data (Cohen & Janicki‐Deverts, 2012), by age groups. Error bars represent standard error of the mean. *p < 0.05; **p < 0.01; †*p* < 0.001

Given that the general population sample (Cohen & Janicki‐Deverts, 2012) was older on average (*M* = 44.6 years, SD = 15.5) than the current study's autistic sample (*M* = 38.5, SD = 13.6), and given that in the general population sample, age was inversely associated with perceived stress (older age associated with lower levels of perceived stress), we conducted a series of post‐hoc one sample *z*‐tests to examine whether the general population and autistic samples differed for mean stress across age groups. We divided our sample into the same six age bins reported on for the general population sample: under age 25, 25–35 years, 35–44 years, 45–54 years, 55–64 years, and aged 65 and older. The one sample z‐tests revealed that for all six age categories, autistic adults reported significantly more perceived stress relative to the general population sample (*z*s ≥2.20, *p*s <0.01). See Figure 2.

Turning our attention to the analyses involving the current study sample only, the 2 × 2 ANOVA within this autistic adult sample revealed a main effect of sex designated at birth for PSS total score, with autistic females reporting higher perceived stress relative to autistic males (autistic females: *n* = 423, *M* = 2.40, SD = 0.71; autistic males: *n* = 290, *M* = 2.12, SD = 0.76; *F*[1709] = 23.58, *p* < 0.001, *ηp* ^2^ = 0.03), but neither a significant main effect of age group (18–39: *n* = 421, *M* = 2.31, SD = 0.75; 40–83: *n* = 292, *M* = 2.25, SD = 0.73; *F*[1709] = 0.84, *p* = 0.36, *ηp* ^2^ = 0.001), nor a significant interaction between age group and sex designated at birth (*F*[1709] = 0.46, *p* = 0.50; *ηp* ^2^ = 0.001).

**FIGURE 3:**
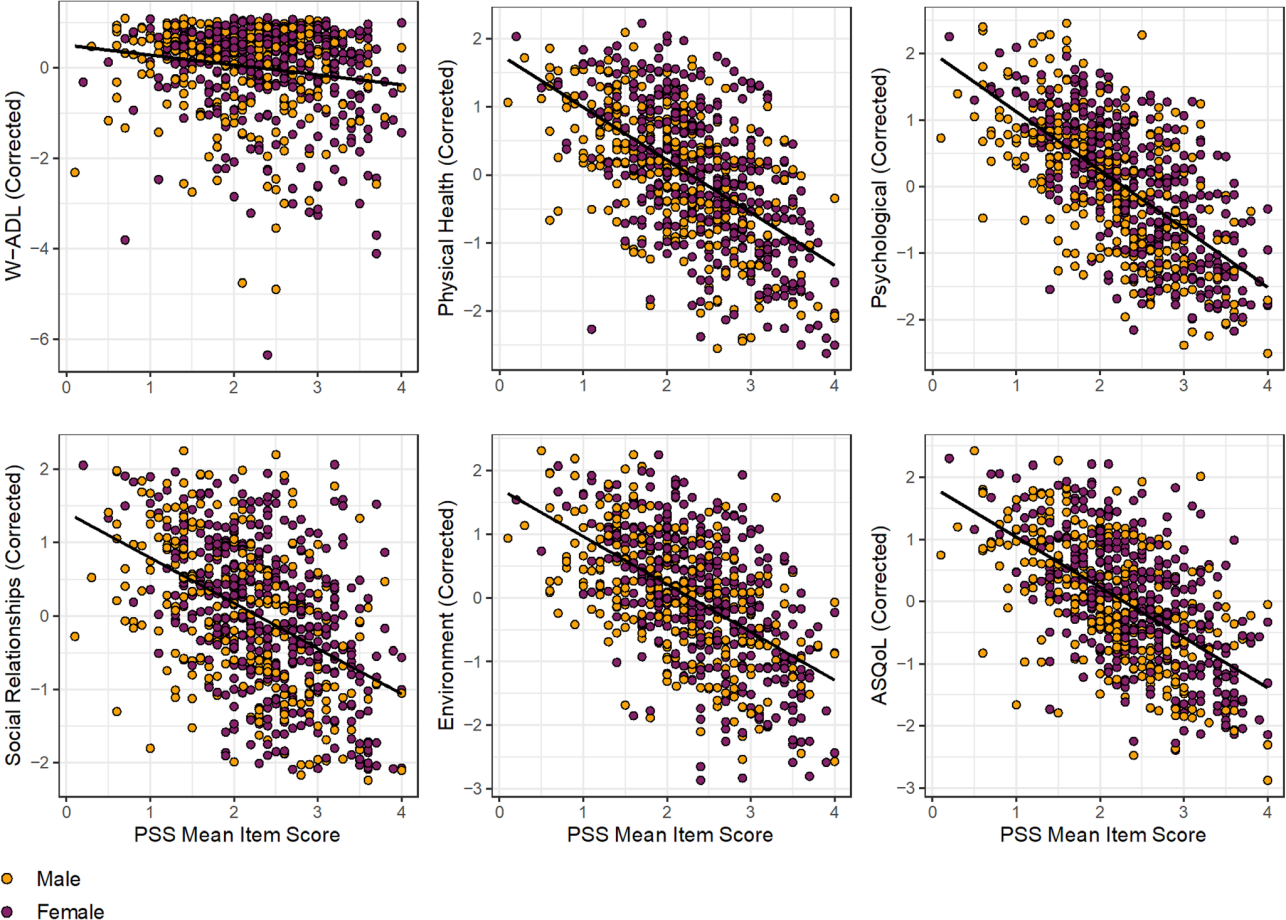
Plots of Perceived Stress Scale (PSS) mean item scores and values for activities of daily living (W‐ADL) and subjective quality of life (WHOQOL‐BREF domains and ASQoL) after regressing out effects of sex designated at birth, age, and total household income in the autistic adults sample

Results of multiple linear regression analyses are reported in Table 3 and Figure 3. An evaluation of covariates revealed that, with the exception of Social Relationships, sex designated at birth contributed significant variance to all domains of subjective QoL, with autistic adults designated female at birth reporting lower subjective QoL compared to those designated male at birth. Age was also a significant predictor in the model in which activities of daily living was the dependent variable, with older age associated with greater independence in activities of daily living. Age also significantly contributed to models of Physical Health, Social Relationships, and Autism‐related QoL, with older age associated with lower subjective QoL in these domains. Household income significantly contributed to all models, with higher household income associated with greater independence in activities of daily living and higher subjective QoL across all five domains.

**TABLE 3:** Quality of life and daily living skills ratings regressed onto age, sex designated at birth, household income, and perceived stress

	W‐ADL					Physical Health QOL					Psychological Health QOL				
	*B*	SE	95% CI	*β*	*t*	*B*	SE	95% CI	*β*	*t*	*B*	SE	95% CI	*β*	*t*
Step 1:	*R* ^2^ = 0.05, *F* = 11.96, *p* < 0.001					*R* ^2^ = 0.11, *F* = 30.24, *p* < 0.001					*R* ^2^ = 0.04, *F* = 8.91, *p* < 0.001				
Age	0.002	0.001	[0.001, 0.004]	0.11	2.96*	−0.007	0.002	[−0.011, −0.002]	−0.11	−2.95*	−0.001	0.002	[−0.005, 0.004]	−0.02	−0.40
Household income	0.02	0.004	[0.011, 0.029]	0.17	4.60**	0.08	0.01	[0.06, 0.11]	0.23	6.38**	0.05	0.01	[0.04, 0.08]	0.15	3.95**
Sex designated at birth	−0.001	0.02	[−0.043, 0.040]	−0.002	−0.06	−0.38	0.06	[−0.51, −0.26]	−0.22	−6.07**	−0.18	0.06	[−0.31, −0.06]	−0.11	−2.84*
Step 2:	Δ*R* ^2^ = 0.03, Δ*F* = 20.60, Δ*p* < 0.001					Δ*R* ^2^ = 0.31, Δ*F* = 385.82, Δ*p* < 0.001					Δ*R* ^2^ = 0.44, Δ*F* = 591.42, Δ*p* < 0.001				
PSS mean score	−0.06	0.01	[−0.09, −0.04]	−0.17	−4.54**	−0.68	0.03	[−0.74, −0.61]	−0.58	−19.64**	−0.78	0.03	[−0.84, −0.71]	−0.69	−24.3**

	Social Relationships QOL					Environment QOL					Autism Specific QOL				
	*B*	SE	95% CI	*β*	*t*	*B*	SE	95% CI	*β*	*t*	*B*	SE	95% CI	*β*	*t*
Step 1:	*R* ^2^ = 0.03, *F* = 7.71, *p* < 0.001					*R* ^2^ = 0.14, *F* = 39.66, *p* < 0.001					*R* ^2^ = 0.11, *F* = 30.01, *p* < 0.001				
Age	−0.01	0.003	[−0.02, −0.006]	−0.16	−4.12**	−0.003	0.002	[−0.008, 0.001]	−0.06	−1.62	−0.01	0.002	[−0.02, −0.007]	−0.18	−5.00**
Household income	0.05	0.02	[0.02, 0.08]	0.12	3.10*	0.12	0.01	[0.10, 0.15]	0.37	10.43**	0.10	0.01	[0.073, 0.12]	0.27	7.60**
Sex designated at birth	0.04	0.08	[−0.11, 0.19]	0.02	0.54	−0.11	0.06	[−0.22, 0.002]	−0.07	−1.94	−0.21	0.06	[−0.33, −0.09]	−0.12	−3.44*
Step 2:	Δ*R* ^2^ = 0.22, Δ*F* = 204.55, Δ*p* < 0.001					Δ*R* ^2^ = 0.28, Δ*F* = 351.35, Δ*p* < 0.001					Δ*R* ^2^ = 0.34, Δ*F* = 440.14, Δ*p* < 0.001				
PSS mean score	−0.66	0.05	[−0.75, −0.57]	−0.48	−14.30**	−0.60	0.03	[−0.66, −0.54]	−0.55	−18.74**	−0.69	0.03	[−0.75, −0.62]	−0.60	−20.98**

* *p* < 0.01.

** *p* < 0.001.

After accounting for the significant effects of sex designated at birth, age, and household income, Bonferroni‐corrected (for the number of regression models evaluated: 0.05/6 = *p* < 0.0083) results revealed that perceived stress contributed significantly to the models for activities of daily living and to all metrics of QoL. Specifically, perceived stress was significantly associated with less independence in activities of daily living (*β* = −0.17, *t* = −4.54, *p* < 0.001, Δ*R* ^2^ = 0.03, adjusted *R* ^2^ = 0.07), and with lower autism‐related QoL (*β* = −0.69, *t* = −20.98, *p* < 0.001 Δ*R* ^2^ = 0.34, adjusted *R* ^2^ = 0.45), as well as lower QoL for all four WHOQOL‐BREF domains: Physical Health (*β* = −0.68, *t* = −19.64, *p* < 0.001 Δ*R* ^2^ = 0.31, adjusted *R* ^2^ = 0.42), Environment (*β* = −0.60, *t* = −18.74, *p* < 0.001 Δ*R* ^2^ = 0.28, adjusted *R* ^2^ = 0.42), Psychological Health (*β* = −0.69, *t* = −24.32, *p* < 0.001 Δ*R* ^2^ = 0.44, adjusted *R* ^2^ = 0.47), and Social Relationships (*β* = −0.48, *t* = −14.30, *p* < 0.001, Δ*R* ^2^ = 0.22, adjusted *R* ^2^ = 0.24). Thus, effect sizes demonstrated small (W‐ADL), medium‐to‐large (WHOQOL Social Relationships), and large (ASQoL and the WHOQOL Physical Health, Psychological Health, and Environment domains) effects of perceived stress on daily living skills and subjective QoL.

## DISCUSSION

The current study aimed to advance the understanding of perceived stress and its potential associations with activities of daily living and subjective QoL in autistic adults. In a large sample, spanning young, middle, and older adulthood, we present robust findings, consistent with the extant literature (Bishop‐Fitzpatrick, Minshew, et al., 2017; Hirvikoski & Blomqvist, 2014; McLean et al., 2021), that autistic adults report higher perceived stress relative to non‐autistic adults, and autistic adults designated female at birth evince higher perceived stress relative to autistic adults designated male at birth (Hong et al., 2016). The findings reported here also augment the literature, showing that after controlling for sex designated at birth, age, and total household income, perceived stress in autistic adults is significantly associated with less independence in activities of daily living and lower subjective QoL across all measured domains—Physical Health, Psychological Health, Environment, Social Relationships, and Autism‐related QoL.

We found autistic adults designated female and male at birth reported significantly elevated perceived stress relative to females and males, respectively, in a large general population sample (Cohen & Janicki‐Deverts, 2012). Furthermore, post‐hoc analyses across six age groups spanning adulthood revealed significantly higher perceived stress scores among autistic individuals compared to those in the same general population sample. Taken together, these results are in accord with research reporting autistic adults show higher levels of perceived stress compared to neurotypical adults (Bishop‐Fitzpatrick, Minshew, et al., 2017; Hirvikoski & Blomqvist, 2014; McLean et al., 2021). Consistent with broader findings on perceived stress in the general population (Cohen & Janicki‐Deverts, 2012), autistic adults designated female at birth reported significantly higher levels of perceived stress compared to those designated male at birth. The sex differences reported in the current study are consistent with a study with a considerably smaller sample of 60 autistic adults (female, *n* = 14), which found that compared to autistic males, autistic females self‐reported greater levels of perceived stress (Hong et al., 2016).

After accounting for the effects of sex designated at birth, age, and total household income, and subsequent to correcting for multiple comparisons, perceived stress contributed significantly to all models: greater levels of perceived stress predicted less independence in activities of daily living and lower subjective QoL. To date, only one other study has examined effects of self‐reported perceived stress on independence in activities of daily living (Bishop‐Fitzpatrick, Minshew, et al., 2017). Unlike the current study, this earlier study, which had a relatively small sample (*n* = 40), did not find that perceived stress contributed significantly to activities of daily living. This inconsistency in findings may be due to power differences between the two studies, as the association between perceived stress and activities of daily living detected in the current study was relatively small in magnitude. One factor that may be contributing to this modest association is a ceiling effect on the W‐ADL in autistic adults without co‐occurring intellectual disability. Consistent with this interpretation, just over a third of participants in the current sample (34%, *n* = 244) scored the maximum value on the W‐ADL.

The current study further found that higher perceived stress was associated with poorer subjective QoL for the ASQoL and all four WHOQOL domains: Physical Health, Psychological Health, Environment, and Social Relationships. These findings converge with literature indicating that greater levels of perceived stress in autistic adults are associated with overall lower subjective QoL (Bishop‐Fitzpatrick et al., 2018; Bishop‐Fitzpatrick, Smith DaWalt, et al., 2017). Distinct from previous studies showing links between perceived stress and a composite metric indexing *overall* subjective QoL in autistic adults, the current study's large sample size permitted us to examine each subjective QoL domain separately. These findings suggest that perceived stress is closely related to subjective quality of life across the board, rather than being related only to selective aspects of quality of life.

Among the strengths of the current study are its statistical power and rigor. The sample size in the current study is greater than 10 times the largest sample size in the literature to date on self‐reported perceived stress in autistic adults. Additionally, the current study's sample was enriched for individuals designated female at birth, an understudied population in autism research: the number of adults designated female at birth in the current study is more than 30 times the largest number of female participants included in the literature on perceived stress in autistic adults. Furthermore, the large overall sample size of the current study permitted the examination of specific domains of subjective QoL, rather than examining overall subjective QoL (as done in the prior studies to date) while controlling for key confounding variables and correcting for multiple comparisons. The sample's composition, spanning young, middle and older adulthood, allowed us to probe for age differences, and the enrichment for autistic individuals designated female at birth afforded statistical power to further probe birth‐sex, and birth‐sex by age interactions. The majority of the limited research to date examining subjective QoL in autistic adults has studied those in early to middle adulthood (Geurts et al., 2021). In contrast, in the current study, 21% of autistic adults were aged ≥50 years, helping to bridge the gap in our knowledge of older autistic adults.

Alongside these numerous strengths, are limitations that should be considered when contextualizing findings. In the current study, participants completed the WHOQOL‐BREF and the ASQoL, but not the WHOQOL Disabilities module (WHODIS; Power et al., 2010). The WHOQOL‐DIS is described as an add‐on module to the WHOQOL‐BREF to assess subjective QoL in adults with physical or intellectual disabilities (Power et al., 2010). The ASQoL, in turn, is described as providing “autism‐specific” items to be used alongside the WHOQOL‐BREF and the WHOQOL‐DIS (McConachie et al., 2018). The decision not to administer the WHOQOL‐DIS was based on both conceptual and practical considerations. Conceptually, the WHOQOL‐DIS is intended for use in adults with intellectual or physical disabilities. The current study included participants SPARK designated as “independent adults,” meaning participants could consent for themselves, and so were unlikely to have a co‐occurring intellectual disability. Additionally, prior to data collection, we did not know whether or how many autistic participants would report a physical disability that would make administration of the WHOQOL‐DIS appropriate. These conceptual considerations were accompanied by the more practical, yet essential, concern of participant burden. The WHOQOL‐BREF and ASQoL were administered as part of a broader battery of surveys investigating adult outcomes in ASD, and the decision to include these measures of subjective QoL but omit the WHOQOL‐DIS also took into consideration the length of the overall survey battery.

The current study also relied on a short self‐report survey to measure perceived stress. To advance and enrich our understanding of perceived stress in autistic adults, future studies should include metrics that capture the lived experience of everyday stressors and stress. For instance, future research might include qualitative interviews, in addition to questionnaire data. Research might also engage autistic adults as research partners to design probes using Cognitive Interviewing Techniques (Beatty & Willis, 2009) to gain knowledge concerning how autistic adults respond to questions on the PSS, and to illuminate key facets of the lived experience of everyday stress and its impacts on activities of daily living and subjective QoL for autistic adults.

The results reported here point to additional important directions for future research. The ceiling effects on the W‐ADL discussed above highlight the need for future studies to examine perceived stress as it may relate to activities of daily living using a measure that is more sensitive to variability in activities of daily living in autistic adults without co‐occurring ID. Further, the potential bi‐directional associations between challenges in certain activities of daily living (e.g., grocery shopping, scheduling appointments by phone) and perceived stress should be examined: the experience of difficulties with certain activities of daily living, along with expectations that the autistic person “should” be able to easily tackle these activities given their cognitive abilities, may itself be a source of stress for some autistic adults without co‐occurring ID.

The current study included only autistic adults without ID, limiting the generalizability of the findings reported here. Thus, studies that include autistic adults with co‐occurring ID are needed to evaluate associations of perceived stress with activities of daily living and subjective quality of life. Another important limitation in the current study is that the sample of autistic adults reported on here was predominantly White (~84%), non‐Latinx (~90%). As such, the lack of representativeness in the sample limits generalizability of the current study's findings, and highlights the need for recruitment of individuals from identities underrepresented in autism research (Giwa Onaiwu, 2020; Jones et al., 2020).

Studies examining the effects of therapeutic interventions on perceived stress (Pahnke et al., 2019; Wijker et al., 2020) provide suggestive evidence that perceived stress may be a modifiable target for intervention. Prior studies have demonstrated associations between perceived stress and subjective QoL (Bishop‐Fitzpatrick et al., 2018; Bishop‐Fitzpatrick, Smith DaWalt, et al., 2017; McLean et al., 2021). The current study strengthens and extends these links. Taken together, the current and prior research suggests that interventions that decrease perceived stress may help improve subjective QoL in autistic adults, although this remains to be empirically verified.

In addition to examining the potential for reducing perceived stress as a way to improve subjective QoL in autistic adults, it is critical to consider *external factors* that plausibly contribute to elevated levels of perceived stress and reduced subjective QoL in autistic adults. For example, autistic individuals may be vulnerable to a range of adverse life events, including bullying (Cappadocia et al., 2012; Weiss & Fardella, 2018), and physical abuse and sexual victimization (Mandell et al., 2005; Ohlsson Gotby et al., 2018). These cumulative experiences of trauma may contribute to elevated mental health difficulties and decreased life satisfaction (Fuld, 2018; Griffiths et al., 2019; Haruvi‐Lamdan et al., 2020; Taylor & Gotham, 2016). Additionally, experiences of minority stress (Meyer, 2003), including social stressors such as stigma and discrimination may increase risk for developing mental health problems in autistic adults (Botha & Frost, 2020; Mitchell et al., 2021). A particularly important consideration for future research is not only the neurominority (i.e., autistic) identity of autistic adults, but also how other minority identities, including diversity in gender identity and sexual orientation, which appear to be more common in autistic persons (Dewinter et al., 2017; George & Stokes, 2017; Warrier et al., 2020; Weir et al., 2021), may increase minority stressors experienced by autistic individuals, and in turn contribute to elevated perceived stress.

To investigate if there was a significant association between perceived stress and both activities of daily living and subjective QoL after accounting for demographic factors known to relate to these key outcomes, the current study controlled for age, birth‐sex, and household income in modeling. Of these demographic factors, we highlight here that household income contributed significantly to all models in that higher income was associated with more independence in activities of daily living and better subjective QoL. As other research has documented that (a) diminished household income is associated with greater disparities in access to employment opportunities among transition‐aged autistic adults (Roux et al., 2015), and (b) autistic adults face high rates of unemployment and underemployment (Shattuck et al., 2012; Taylor & DaWalt, 2017; Taylor & Seltzer, 2011), socioeconomic status (as estimated here via report of household income) may directly or indirectly (e.g., via employment) play a role in experiences of perceived stress and its association with activities of daily living and subjective QoL among autistic adults. However, empirical work is largely absent in this area. The results presented here in concert with the larger literature on employment, socioeconomic status, and ASD suggest that more attention should be paid to the role socioeconomic status plays in the well‐being of autistic adults. More broadly, future work is needed that examines higher perceived stress and lower subjective QoL not simply within the autistic individual but rather as sequelae embedded within a broader context of individual‐environment interactions.

## Supporting information


**Data S1.** Supporting Information.

## Data Availability

Qualified researchers approved by SPARK can obtain the broader demographic and phenotypic data described in this study by applying through SFARI Base at https://base.sfari.org/.
