# Supplementary material for: Increased perceived stress is negatively associated with activities of daily living and subjective quality of life in younger, middle, and older autistic adults
Source: Autism Res. 2022 Jul 5;15(8):1535–49. doi: 10.1002/aur.2779 (PMC9545671; doi:10.1002/aur.2779)
Supplement: Supplementary file 1 — Data S1. Supporting Information. [file AUR-15-1535-s001.docx]

**Supplemental Methods**

As indicated in the Methods section of the main text, the regression between perceived stress and Psychological Health QoL was rerun with a single item from the Psychological Health QoL domain (“How often do you have negative feelings, such as blue mood, despair, anxiety, depression?") omitted. This item was omitted due to its content overlap with an item on the PSS (“In the last month, how often have you felt nervous and stressed?”), in order to confirm that findings remained unchanged by this item’s omission.

**Supplemental Results**

After rerunning the regression examining links between perceived stress and Psychological Health QoL with the single item from the Psychological Health QoL domain referenced above omitted due to its content overlap with a PSS item, findings for this model remained substantively unchanged. Specifically, after removal of this item, and after accounting for effects of sex designated at birth, age, and household income, perceived stress was significantly associated with lower Psychological Health (β=-0.64, *t*=-21.32, *p*<.001 ΔR^2^=.38, adjusted R^2^=.41).
